# Screening Tests of Reproductive Immunology in Systemic Lupus Erythematosus

**DOI:** 10.1155/2012/812138

**Published:** 2012-10-24

**Authors:** Zdenka Ulcova-Gallova, Alice Mockova, Miroslava Cedikova

**Affiliations:** ^1^Department of Obstetrics and Gynecology, Faculty of Medicine in Pilsen and University Hospital, University in Pilsen, 30460 Pilsen, Czech Republic; ^2^Department of Neonatology, Faculty of Medicine in Pilsen and University Hospital, Charles University in Prague, Alej Svobody 80, 30460 Pilsen, Czech Republic; ^3^Institute of Histology and Embryology, Faculty of Medicine in Pilsen, Charles University in Prague, Karlovarská 48, 30166 Pilsen, Czech Republic

## Abstract

Female patients in reproductive age with systemic lupus erythematosus and fertility complications together are observed by rheumatologists, gynecologists, and reproductive immunologists. The paper notes the presence of autoantibodies to zona pellucida, to phospholipids (phosphatidyl serine, phosphatidyl ethanolamine, phosphatidyl inositol, phosphatidyl glycerol, phosphatidic acid, annexin V, beta-2 glycoprotein I, and cardiolipin) and of isoantibodies to sperm cells. Isoantibodies to sperm cells are not significantly predominant, but autoimmunity is well expressed in IgG positivity against phosphatidyl inositol, phosphatidyl ethanolamine, phosphatidyl serine, cardiolipin, and beta-2 glycoprotein I, as well as antizona pellucida antibodies in IgG isotype. According to the levels of autoantibodies we have to choose preventive treatment to protect mother and her foetus.

## 1. Introduction

Although autoimmune diseases (AIDs) are not in general considered a major cause of impaired reproductive capacity some AIDs are associated with infertility or pregnancy wastage. Autoimmune conditions may affect all stages of fertility, from ovarian to implantation failure and pregnancy loss. Systemic lupus erythematosus (SLE) is one of the most common autoimmune disorders that affect women during their childbearing age. Prevalence of SLE in Europe is similar as in USA, ranging from 3.3 to 4.8 per 100.000 person-years [1].

Typical clinical symptoms of SLE include fatigue, fever, arthritis, a photosensitive rash serositis, Raynaud phenomenon, glomerulonephritis, vasculitis, and hematologic abnormalities. Although patients with SLE are as fertile as women in the general population, their pregnancies could be associated with complications [2]. Fertility may be compromised by menstrual irregularities with anovulatory cycles during episodes of active disease or chronic renal failure, use of nonsteroid anti-inflammatory drugs (NSAIDs), high-dose corticosteroids, or cyclophosphamide.

High rate of fetal losses up to 45 percent in SLE women has been described in some studies [2]. Another complication may be implantation failure after in vivo fertilization and embryo transfer or impaired fetal development.

There is a wide variety of autoantibodies associated with SLE. Some of the antibodies are helpful in the diagnostics of the illness, while others are more useful in detecting and monitoring disease activity or potential complications. Antibodies to native double-strand DNA (dsDNA) are relatively specific for the diagnosis of SLE. Serum antinuclear antibodies (ANAs) are found in nearly all individuals with active SLE. Significant for SLE diagnosis is assessment of spectrum extractable nuclear antibodies (Sm, La, Ro), antibodies to ribonucleoprotein (RNP), complement and N-methyl-D-aspartate receptor (ENA panel).

Antiphospholipid antibodies (aPLs) form a large group of antibodies that are detected in patients with SLE as well as with other autoimmune conditions. These antibodies are associated with a wide range of potential complications during pregnancy, including miscarriage, fetal death, intrauterine growth restriction, prematurity, and preeclampsia-especially in the primary antiphospholipid syndrome (APS).

In SLE women, pregnancy should be best planned during periods of disease stabilization and nephritis remission lasting at least six months. Closed collaboration of rheumatologist, obstetrician, and neonatologist is necessary for successful pregnancy and delivery. Fulfilling these prerequisites, there is still around 5% of women with SLE that have fertility or pregnancy problems [3].

 A complication in reproduction is very often a reason for thorough immunological examination. We present here our experience with SLE patients from the perspective of reproductive immunology.

## 2. Objectives

The general aim of our paper is to evaluate the results of screening tests in reproductive immunology in women of childbearing age with SLE. The primary aim is to investigate the occurrence of autoantibodies to zona pellucida and eight various phospholipids and occurrence of isoantibodies to sperm cells in patients with SLE in remission planning pregnancy. 

## 3. Subjects

The study group consists of 52 woman with SLE (mean time from diagnosis 4.62 ± 2.28 years; age 18–42 years, mean 30.4 ± 3.9) that were referred for pregnancy planning to Special Division for Infertility and Immunology of Reproduction at the Department of Obstetrics and Gynaecology, Charles University and Faculty Hospital, Pilsen, Czech Republic. All patients fulfilled the revised criteria for SLE diagnosis [4]. All patients were in remission during the study examinations and during attempts to fertilize, none had acute nephritis or serious renal impairment. None of the patients were treated with corticosteroids in a dose exceeding 10 mg per day, immunosuppressive drugs, only three were on hydroxychloroquine sulfate (plaquenil) medication. Before being examined and treated at our Division, all patients were examined by endocrinologist, gynecologist, and had genetic consultation. No substantial pathology was found in the following hormonal tests that are known to impair fertility: FSH, LH, progesterone, estradiol, prolactin. Pregnancy loss occurred in 38 women from the study group prior to our testing.

The control group consisted of 25 age-matched healthy fertile women (age 24–43 years, mean 31.2 ± 3.2). All control subjects have successfully conceived and have given spontaneous birth to two healthy children, they had prior to our examination regular sexual intercourse and menstrual cycle and did not use hormonal contraceptives. The study was approved by the local research ethics committee at the Charles University Medical Faculty Hospital, Pilsen, and written informed consent was obtained from all participants.

## 4. Methods

Detection of autoantibodies to zona pellucida, eight phospholipids (phosphatidyl-serine, phosphatidyl-ethanolamine, phosphatidyl-inositol, DL-glycerol, phosphatidic acid, annexin V, beta-2-glycoprotein I, and cardiolipin), and isoantibodies to sperm cells was performed as described below.

Both groups were tested with *F*-test of equality of variances. For each immunology test percentage values were calculated independently in study and control group, and antibody occurrences were analyzed using standard odds ratio formulas where applicable.

### 4.1. Sperm Antibodies of Serum (ASA)

We have performed tray agglutination test (TAT) [2] and indirect-mixed agglutination test (i-MAR) according to previously published guidelines for examination of the serum ASA [3]. TAT test serves as an initial screening examination. The blood from studied subjects was collected by venipuncture into evacuated tubes and centrifuged. Isolated serum was inactivated by heat (56°C for 30 min) and stored at −20°C until analysis. We performed the TAT test by adding 5 *μ*L of inactivated, geometrically diluted serum and 1 *μ*L of motile donor sperm (40·10^6^/mL), isolated by “swim-up technique” into microchambers covered by paraffin oil. After the incubation (2 h at 37°C), the immunological reaction was evaluated under inverted microscope at 200x magnification. Agglutination of the sperm cells at dilution at least 1 : 64 was considered as a positive result.

The i-MAR test was performed to analyze the antisperm response in IgG and IgA class. One microliter of native sperm suspension, 1 *μ*L of inactivated serum, and 1 *μ*L of glutaraldehyde-fixed sheep erythrocytes precoated with human IgG and IgA were mixed together. Then, 1 *μ*L of the corresponding antiserum anti-IgG and anti-IgA (Behringer, Hannover, Germany) was added. Finally, the mixture was covered with coverslip and incubated in humid Petri chamber for 5–10 min. The result of the sperm agglutination reaction was watched under the inverted microscope at 200x magnification. The i-MAR test was considered as positive, if more than 49% of motile spermatozoa were involved in mixed agglutinates (spermatozoa and sheep erythrocytes coated by the corresponding immunoglobulin).

### 4.2. Sperm Antibodies in Cervical Ovulatory Mucus

We use ovulatory cervical mucus taken by a special syringe 5 days after condom protected intercourse that is planned to happen during ovulation time. Sperm capillary penetration test serves as screening test, and i-MAR test detects local sperm antibodies of IgG and/or IgA [4]. 

### 4.3. Zona Pellucida Antibodies

Classic ELISA tests (Laboserv) for detection of zona pellucida antibodies in isotype of IgG and IgM were used.

### 4.4. Panel of Antiphospholipid Antibodies (aPL)

ELISA was also used for detection of aPL against phosphatidyl serine, phosphatidyl ethanolamine, phosphatidyl inositol, DL-glycerol, and phosphatidic acid. For ELISA, polystyrene microtiter plates were coated with 50 *μ*L of phospholipid (50 *μ*L/mL of methanol) and were allowed to dry overnight at 4°C. The plates were then blocked by solution containing 10% of fetal calf serum (FCS) in 1 M TRIS buffer solution (TBS—250 mM Tris, 1.5 M NaCl), for 30 min at room temperature. Subsequently, polystyrene microtiter plates were washed three times by binding buffer (Na_2_CO_3_, NaHCO_3_, NaN_3_, pH 9.6).

Serum from patients and controls, diluted to a ratio of 1 : 50 in TBS containing 10% FCS were added to the wells after the wash. The plates were incubated for 2 hours at room temperature and then washed five times with TBS containing 0.05% Tween 20. Fifty microliters of peroxidase-conjugated antihuman immunoglobulin (IgG, IgA, or IgM) was added and incubated for 1 hour at room temperature. The plates were then washed five times in TBS Tween. Fifty microliters of substrate solution were added to each well and incubated in the darkness for 30 min at room temperature. The reaction was stopped by adding 50 *μ*L of 2 M sulphuric acid. The optical density of each well was determined using a Titertek MultisKan MCC/340 (Flow Laboratories, London, UK) at 492 nm. Background optical density was run for all patients' sera diluted 1 : 50 in wells on identical microtiter plates coated with methanol but without phospholipids. The phospholipids used to coat the ELISA plates were: phosphatidyl serine, phosphatidyl ethanolamine, phosphatidyl inositol, DL-glycerol, and phosphatidic acid (Sigma, St.Louis, MO, USA). 

For detection and quantification of serum antiannexin V (Szabo-Scandic Handels GMBH & Co KG, Vienna, Austria) commercial ELISA kits were used, as well as for detection of IgG and IgA antibodies against beta2-glycoprotein I (beta2GPI) (Immunotech, Prague Division, Czech Republic), cardiolipin levels in IgG and IgM (Millenia, London, UK) (Table 1). Statistical analysis with the use of Statgraphics software was performed to obtain cut-off levels for Ig isotypes of aPLs that were derived as values above 3 standard deviations (SDs).

## 5. Results

Because equality of variance test proved that both groups were similar in age (as an independent baseline characteristic), we were able to assess individual antibody prevalence percentages as follows: nonzero prevalences of tested antibodies were all higher in the study group compared to controls with the exception of antiphosphatidyl inositol IgM and antiphosphatidyl serine IgM (3.8% versus 4.0% and 3.8% versus 8.0%, resp.). Difference between study group and control group in antisperm antibodies (ASAs) predominance was only nominal and statistically insignificant in both serum ASA (9.6% versus 4.0%, odds ratio 2.55, 95% confidence interval 0.28–23.10) and cervical ovulatory mucus ASA (11.5% versus 4.0%, OR 3.13, 95% CI 0.36–27.52). Concerning antizona pellucida (aZP) and antiphospholipid (aPL) antibodies, no significant difference was observed in IgM subtypes across all tests. However, in aZP IgG subclass a difference was found to be significant in favor of study group (12 out of 52 subjects, 23.0%, versus zero of 25 subjects). The same was true for almost all individual aPL antibodies in IgG isotype, the exception being antiphosphatic acid IgG and antiphosphatidyl glycerol IgG (detailed results are shown in Table 1 with significant values marked in bold script). Even though we detected anticardiolipin IgG positivity in two of the patients from control group (unlike any other IgG in this study), the difference in prevalence between groups (30.8% in study group versus 8.0% in controls) still yielded statistically significant result (odds ratio 5.11, 95% CI 1.07–24.33).

## 6. Discussion

Pregnancy is the most significant exception to the immunological rules where the mothers body not only tolerates, but also broadly supports semiallogenic blastocyst, embryo, and fetus. Remarkable tolerance is provided by complex immunoregulatory mechanisms between the mother and fetal trophoblast. Failure of this tolerance is one of the most common causes of fetal loss. As mentioned in many papers, reproductive failure including recurrent pregnancy loss (RPL) and infertility has been linked to presence of various autoantibodies [5]. 

In SLE as well as in other autoimmune diseases an overproduction of autoantibodies is described, especially against organ-nonspecific antigens. This feature is based on the loss of B-cell tolerance to antigens, nucleic acids, and their binding proteins. Antinuclear antibodies can trigger proliferation of autoreactive lymphocytes in genetically susceptible individuals under the influence of environmental factors (infections, drugs, toxins, smoking, and hormonal factors) leading to expansion of their specificity towards more nuclear antigens or other structures (elements of blood, plasma components, coagulation factors, and complement cascade) [5]. Influenced by many proinflammatory cytokines and chemokines inflammatory cells migrate into target organs, activate their effector mechanisms, and cause chronic inflammation with the help of chronic humoral components.

The aim of our study was to evaluate the occurrence of variety of antibodies possibly responsible for immunological reproduction failure in women with SLE and compare these results with findings in healthy controls.

Many retrospective and prospective studies have proven effect of antisperm antibodies in various human body fluids (serum, semen, cervical ovulatory mucus, follicular, or peritoneal fluid) on fertility of women and men [6]. ASA can influence the mechanisms of transport of spermatozoa within the female genital tract due to decreasing sperm motility, may alter sperm capacitation or the acrosome reaction, can interfere with egg fertilization, or have postfertilization effects on the zygote and preimplantation early embryo [6].

It seems clear that repeated sperm exposition is not sufficient for most women to start production of antisperm antibodies. Other factors such as mechanical, chemical, or inflammatory lesions of the mucosa in female genital system play an important role [7]. Especially in SLE women the immunosuppressive therapy might affect the likelihood of genital tract infection. The proven higher incidence of CMV, EBV virus, and *Chlamydia trachomatis* infection in SLE patients may contribute to their infertility [8, 9]. 

Antisperm antibodies (ASAs) in both of our groups were determined to evaluate the difference. In contrast to the above mentioned facts we have found no significant difference between antisperm antibody prevalence in study group and control group. Our prevalence data from control group are in consent with evidence about the presence of these antibodies in healthy women with undisturbed reproductive capability (several authors reported that these antibodies occur in approximately 1 to 2.5% of fertile men and in 4% of fertile women) [6]. Even though our data may suffer from low sample size bias, the benefit of both serum and cervical ovulatory mucus antibody tests seems to be the lowest in our pool of patients and controls.

Zona pellucida (ZP), ten micrometers “strong” oogenetic glycoprotein matrix, is formed during oogenesis and its thickness increases with the growth of the oocyte. It serves basically as a protective layer surrounding the oocyte as it matures and is composed of three major glycoproteins ZP1, ZP2, and ZP3. These glycoproteins seem to be the important ovarian antigens participating in the etiology of some infertility disorders, including autoimmune premature ovarian failure (POF). POF can be seen in approximately 1-2% of healthy women, in 30% is connected with some autoimmune causes and is often presented in women with SLE [10]. Autoimmune POF has been proven in animal studies, where the preantral mouse follicles with both anti-ZP2 and anti-ZP antibodies were cultured and thereafter have shown smaller diameter than controls [11]. Furthermore, antizonal antibodies against glycoproteins of ZP are able to inhibit sperm attachment and penetration into oocyte and may be the cause of natural or artificial fertilization failure. The increased levels of serum AZA are very often detected after repeated unsuccessful IVF as we reported earlier [12]. Some studies confirmed an antigamete antibodies (AGAs) in high percentage of patients with unexplained infertility versus patients with proven etiology of infertility [13]. 

In the present study we have found borderline significant prevalence of aZP IgG antibodies in the study group in comparison with the control group. 

 Antiphospholipid antibodies (aPL) form a group of antibodies that is probably associated with compromised fertility not only in SLE women. Although some retrospective studies reported nonsignificant association between antiphospholipid antibodies and pregnancy loss in patients with SLE other studies have confirmed statistically significant interrelation between them [14]. APL could be present in up to 5% of apparently healthy controls and up to 37% of patients with SLE. Less frequently they also accompany other autoimmune diseases [15, 16]. In animal models the administration of aPL during pregnancy causes placental impairment followed by miscarriage. The thrombosis in uteroplacental vasculature is then the result of endothelial cell activation, inhibition of protein C/S system and fibrinolysis, and annexin Vdisplacement. Another significant mechanism for nonthrombotic fetal damage is straight influence of aPL on the anionic phospholipids and *β*2-glycoprotein I of trophoblast that very probably impairs the placentary production of chorionic gonadotropin during the early phases of pregnancy [17]. Although the current classification for the antiphospholipid syndrome is based on positive identification of one or more of three standardized laboratory assays: anticardiolipin antibodies (aCL), lupus anticoagulant (LA), and anti *β*(2)glycoprotein I (anti-*β*(2)GPI) according to the report of a task force and preconference workshop at the 13th International Congress on antiphospholipid antibodies, Galveston, Texas in April 2010 several other antibodies have been proposed to be relevant to APS. In our laboratory we use the expanded scale of aPL detection of antiannexin V, antiphosphatidylserine, antiphosphatidylethanolamine, antiphosphatidylinositol, antiphosphatidylglycerol, and antiphosphatidic acid. Our results suggest that in women with SLE the aPL IgM screening is unsatisfactory. On the other hand, IgG screening shows a marked difference that may well be associated with reproductive failure. The observed integrity (5 of 7 aPL IgG antibodies are significantly different between study and control group) may be due to hypothetical associations of certain IgG antibodies resulting in notional fixed antibody profiles that carry sufficient potential for infertility or pregnancy wastage only in cooperation. 

A review of 10 studies of 554 women with SLE found that fetal demise was more common in those with aPL (38 to 59 percent versus 16 to 20 percent in those without such antibodies), LAs (36 versus 13 percent), or aCL (39 versus 18 percent). Fetal loss typically occurs after 10 weeks gestation [5, 18].

The relationship between autoimmunity and reproduction has long been recognized. Although in one critical review about the impact of abnormal autoimmunity on female fertility doubts about influence of aPL to POF in SLE were expressed SLE remains the autoimmune disease mostly compromised by humoral changes. It seems possible that another unexplained mechanism (e.g. presence of other antibodies as an IgG antilaminin-I antibodies, anticorpus luteum glycoprotein and antilymphocytotoxic autoantibodies, or antimitochondrial M5 type antibodies) could be involved [5]. These antibodies were found to interfere with markers of placental and yolk sac differentiation, invasion, endocytosis, signaling, and others promote thrombotic processes [19, 20].

## 7. Conclusion

Based on our results and long-term experience, we believe that it is appropriate in women with SLE with recurrent and otherwise unexplained fetal loss to recommend a detailed evaluation by a specialist in reproductive immunology. In the case of positive findings of autoantibodies, preventive treatment is to be considered (e.g. corticosteroids, low molecular weight heparin, low dose of aspirin, intravenous immunoglobulin therapy). Because the evidence supporting association of various individual antibodies with reproductive failure is inconclusive a new meta-analysis of antibody profiles in women at risk may be of benefit. 

## Figures and Tables

**Table 1:** Prevalence of positive results for each performed antibody analysis with comparison between study and control groups.

Antibody	Number of positive subjects		Odds ratio (95% confidence interval)
	Study group (*n* = 52)	Control group (*n* = 25)	
Antisperm antibodies (ASAs)			
Serum ASA	5/52 (9.6%)	1/25 (4.0%)	2.55 (0.28–23.10)
Cervical ovulatory mucus ASA	6/52 (11.5%)	1/25 (4.0%)	3.13 (0.36–27.52)
Antizona pellucida antibodies (aZP)			
aZP IgG	**12/52 (23.0%)**	**0/25 (0.0%)**	N/A
aZP IgM	1/52 (1.9%)	0/25 (0.0%)	N/A
Antiphospholipid antibodies (aPL) against			
phosphatic acid IgG	1/52 (1.9%)	0/25 (0.0%)	N/A
phosphatic acid IgM	0/52 (0.0%)	0/25 (0.0%)	N/A
phosphatidyl glycerol IgG	2/52 (3.8%)	0/25 (0.0%)	N/A
phosphatidyl glycerol IgM	0/52 (0.0%)	0/25 (0.0%)	N/A
phosphatidyl inositol IgG	**35/52 (67.3%)**	**0/25 (0.0%)**	N/A
phosphatidyl inositol IgM	2/52 (3.8%)	1/25 (4.0%)	0.96 (0.08–11.12)
phosphatidyl ethanolamine IgG	**16/52 (30.8%)**	**0/25 (0.0%)**	N/A
phosphatidyl ethanolamine IgM	3/52 (5.8%)	1/25 (4.0%)	1.47 (0.15–14.88)
phosphatidyl serine IgG	**32/52 (61.5%)**	**0/25 (0.0%)**	N/A
phosphatidyl serine IgM	2/52 (3.8%)	2/25 (8.0%)	0.46 (0.06–3.47)
cardiolipin IgG	**16/52 (30.8%)**	**2/25 (8.0%)**	**5.11 (1.07–24.33)**
cardiolipin IgM	4/52 (7.7%)	0/25 (0.0%)	N/A
beta-2-glycoprotein IgG	**15/52 (28.8%)**	**0/25 (0.0%)**	N/A
beta-2-glycoprotein IgA	9/52 (17.3%)	0/25 (0.0%)	N/A
annexin-V	7/52 (13.5%)	0/25 (0.0%)	N/A

Note: statistically significant values are marked in bold script.

## References

[1] McCarty DJ, Manzi S, Medsger TA, Ramsey-Goldman R, LaPorte RE, Kwoh CK (1995). Incidence of systemic lupus erythematosus: race and gender differences. *Arthritis and Rheumatism*.

[2] Andrade R, Sanchez ML, Alarcón GS (2008). Adverse pregnancy outcomes in women with systemic lupus erythematosus from a multiethnic US cohort: LUMINA (LUI). *Clinical and Experimental Rheumatology*.

[3] Hickman RA, Gordon C (2011). Causes and management of infertility in systemic lupus erythematosus. *Rheumatology*.

[4] Petri M (2005). Review of classification criteria for systemic lupus erythematosus. *Rheumatic Disease Clinics of North America*.

[5] Shoenfeld Y, Blank M (2004). Autoantibodies associated with reproductive failure. *Lupus*.

[6] Chiu WWC, Chamley LW (2004). Clinical associations and mechanisms of action of antisperm antibodies. *Fertility and Sterility*.

[7] Mazumdar S, Levine AS (1998). Antisperm antibodies: etiology, pathogenesis, diagnosis, and treatment. *Fertility and Sterility*.

[8] Febrônio MV, Pereira RMR, Bonfá E, Takiuti AD, Pereyra EAG, Silva CAA (2007). Inflammatory cervicovaginal cytology is associated with disease activity in juvenile systemic lupus erythematosus. *Lupus*.

[9] Berkun Y, Zandman-Goddard G, Barzilai O (2009). Infectious antibodies in systemic lupus erythematosus patients. *Lupus*.

[10] Forges T, Monnier-Barbarino P, Faure GC, Béné MC (2004). Autoimmunity and antigenic targets in ovarian pathology. *Human Reproduction Update*.

[11] Calongos G, Hasegawa A, Komori S, Koyama K (2009). Harmful effects of anti-zona pellucida antibodies in folliculogenesis, oogenesis, and fertilization. *Journal of Reproductive Immunology*.

[12] Ulcova-Gallova Z (2010). Immunological and physicochemical properties of cervical ovulatory mucus. *Journal of Reproductive Immunology*.

[13] Koyama K, Hasegawa A, Tsuji Y, Iosjima S (1985). Production and characterization of monoclonal antibodies to coss-reactive antigens of human and porcine zonae pellucidae. *Journal of Reproductive Immunology*.

[14] Kalunian KC, Peter JB, Middlekauff HR (1988). Clinical significance of a single test for anti-cardiolipin antibodies in patients with systemic lupus erythematosus. *American Journal of Medicine*.

[15] Cervera R, Piette JC, Font J (2002). Antiphospholipid syndrome: clinical and immunologic manifestations and patterns of disease expression in a cohort of 1,000 patients. *Arthritis and Rheumatism*.

[16] Ginsberg JS, Brill-Edwards P, Johnston M (1992). Relationship of antiphospholipid antibodies to pregnancy loss in patients with systemic lupus erythematosus: a cross-sectional study. *Blood*.

[17] Tincani A, Spatola L, Cinquini M, Merino P, Balestrieri G, Shoenfeld Y (1998). Animal models of antiphospholipid syndrome. *Revue du Rhumatisme*.

[18] McNeil HP, Chesterman CN, Krilis SA (1991). Immunology and clinical importance of antiphospholipid antibodies. *Advances in Immunology*.

[19] Cervera R, Balasch J (2008). Bidirectional effects on autoimmunity and reproduction. *Human Reproduction Update*.

[20] Carp HJA, Selmi C, Shoenfeld Y (2012). The autoimmune bases of infertility and pregnancy loss. *Journal of Autoimmunity*.

